# On the Evils of Procrastinating the Operation, in Certain Cases of Strangulated Hernia

**Published:** 1825-06

**Authors:** T.W. Chevalier

**Affiliations:** Surgeon.


**Art. IV.** — *On the Evils of Procrastinating the Operation, in certain Cases of Strangulated Hernia.* By T. W. Chevalier, Esq. Surgeon.

The slighter the operation for the relief of strangulated hernia may be in itself, and in its probable consequences, the less considerable will be the objections against it; and the more decided and the more general the preference that it deserves over any other means by which relief in such cases may be sought.

The more unpromising any other means may be, the greater is the chance of their failure; and of their employment becoming the occasion of the continuance of those circumstances which seemed to indicate their use.

The evils to be expected from the continuance of those circumstances, therefore, together with any that may result from the nature of the remedies employed, are to be weighed against the objections to the operation, when we would decide whether it should be immediately performed, or whether it should be postponed for the purpose of attempting reduction by those remedies.

It is the object of this paper to afford, from such facts as have come under the author's own cognisance, a just estimate of the dangers attendant upon the operation for the relief of all ordinary cases of strangulated hernia; and to compare this estimate with the dangers, both of strangulation itself, and of the means commonly employed in aid of the taxis; and, lastly, to point out some cases, wherein it is respectfully submitted to the consideration of the profession, whether the small encouragement afforded to expect reduction by the taxis be sufficient to justify its continued trial, with the prospect of such delay and danger as its employment, accompanied by its debilitating auxiliaries, is more likely than not to occasion.

The opening made by the trocar, in tapping the abdomen, is a punctured wound: it is made by a coarser instrument, and it is a greater injury to the peritoneum, than that which needs commonly to be made, at the bottom of a free opening, by the bistoury, in the operation for strangulated hernia. The opening made by the trocar in paracentesis abdominis has been many times known to remain open for some days, and air has been admitted into the cavity of the peritoneum; and yet, in most of such cases, no bad consequence has ensued. The admission of air into the "circumscribed cavity" of the peritoneum rarely needs to occur in the operation for hernia; and, where it does happen, it is scarcely ever found to protract recovery, or to render it less regular: and, indeed, it is very questionable whether the intrusion of a small quantity of pure air into a cavity, where it is not liable to produce chemical change, be ever a serious evil.

Whatever may have been the cause of strangulation, properly so called,* it can scarcely be sufficiently complete, or have existed sufficiently long, to render reduction difficult, if even to have excited any alarm in the mind of the patient, without some degree of inflammation already excited at the place of the stricture, or, it may be, for a considerable extent around it. This inflammation, uncertain in its degree, arises from a cause presently to be more fully considered; but it must be here observed, that the author has almost invariably seen it alleviated for a time, if not permanently assuaged and removed, by the operation.

In the operation for tying the external iliac artery, the peritoneum, although not opened, is torn from its attachments and exposed, and not very gently handled, and this through a considerable extent; and there have, nevertheless, been several recoveries, without peritoneal inflammation. The peritoneum, situated as such, is not exposed in the operation for hernia; but the hernial sac, a peritoneal production or process, is laid open; and it is to be considered whether the objections to the operation are not founded upon this exposure, as well as that of the strangulated intestine? It would appear that they are so; and in this view they are to be estimated,—not, however, before it has been considered what other circumstances predispose patients to them. For it is certainly true (unless the author has much deceived himself,) that where the operation for hernia has been performed, before any other symptoms had come on, than those merely which are essential to the strangulation of an intestine, the wound has healed, and the patient has got well, without any unfavourable effect of the operation! and hence it is to be presupposed that, where the operation has been followed by unfavourable symptoms, they are attributable chiefly, or at least greatly, to some other predisposing circumstances, rather than to the operation itself.

When a patient finds that his rupture will not go back, that he has no evacuation, and that the hernial tumor is becoming tender, he immediately suspects the consequences; and, upon finding the surgeon as unable as himself to accomplish reduction, he becomes confirmed in the opinion that the continuance of his present state is inconsistent with life. At the same time the intestines above the hernia are becoming more and more

* The term *strangulation* has been properly restricted to those wherein the circulation in the blood-vessels of the protruded portion of intestine is obstructed, or considerably impeded, by means of the stricture upon it; the term *incarceration* implying a mechanical impediment by the same means, but merely to the passage of the intestinal contents. Where less than the whole calibre of the gut is *strangulated*, one can scarcely say with propriety that the intestine is *incarcerated*; so that strangulation and incarceration may exist, either without the other: and I have seen each without the other, producing death, by sloughing of the intestine above the stricture.

loaded; their ordinary circumstances, viz. *those of a pendulous, freely moveable, and pervious canal*, are completely changed; an injury is done to that part of the bowels which is within the stricture; and a condition most palpably opposite to nature,—a condition wherein the course of the contents of the alimentary canal is inverted, is impressively and sensibly portended.

It may be that the patient is also in pain; it may be that one of those diseases which most violently prostrate the strength, that peritoneal inflammation, has begun, and has arisen from a cause which its usually proper treatment cannot relieve; so that every thing is tending to debilitate the patient, and to benumb the restorative powers of his system; while a part of one of his vital organs is threatened with mortification, or gangrene has already commenced, and the conflict is extreme.

I have seen a woman who had no strikingly urgent symptoms twenty-four hours after her hernia had come down, and been strangulated: she had, however, an expression of countenance that made one feel that her case was serious, and a determined opinion that she should die. The operation was performed, as is said, only twenty-four hours from the commencement of strangulation, and the gut was found to have been opened largely into the sac by gangrene; and the next day she died.

The consequences of the continuance of such a condition as has been described to be that of a patient labouring under strangulated hernia, or even of those circumstances which forbode it, are in some instances referable *only to that continuance.* For example, in those cases where dividing the stricture and releasing the gut neither aggravates nor alleviates the symptoms, but where they go on as they would have gone on had no operation been performed;—in those cases where the operation fails to re-establish the bowel in the due performance of its functions, and where the gut, although released, will not transmit the intestinal contents;—in those cases where the operation fails to retard, or to prevent, the progress of ulceration and the access of gangrene;—in those where the patient speedily dies, without any obvious cause; or rather, perhaps, it should be said without any other ostensible cause than the delay of the operation;—and possibly in other cases also besides those which I have here enumerated, as having myself seen them.

Often, however, there occurs serious symptoms after the operator has relieved strangulation, which (notwithstanding the prostration of strength that it occasions, and the debilitating influence of violent sudorifics, nausea, and blood-letting, used in aid of the taxis,) are not exclusively referable to the continuance of strangulation, nor so indubitably its consequence. Where, for example, the symptoms produced by the continuance of strangulation have been relieved by the operation; and where it is from twelve to twenty-four hours afterwards that the same, or similar, symptoms recur; or where no serious symptoms existed previously to the performance of the operation, and where yet they come on afterwards.

In such cases, what degree of inflammation existed around the stricture at the time of the operation? what degree of pyrexial symptoms? or what degree of that disposition to asthenic pyrexia, which is the direct result of weakness, were to be observed at the time of the operation? Were not the patient's restorative powers, together with the morbid energy of his circulatory and nervous systems, then benumbed, and (until the delayed operation, to a certain extent, relieved them,) too inert, too oppressed, to exhibit the danger with which they were then pregnant; and by which they now subdue the still feeble, though liberated, sufferer? In short, is the inflammation subsequent to the operation, in any degree, the effect of the wound and of the exposure? If it were so, it should manifest some accordance with the state of the wound; it should bear some direct proportion to the severity of the operation performed; it should always originate at the wound, or spread from it as from a centre or source; it should affect the peritoneum of the parietes in particular, or that of the handled intestine, in proportion as either has been more roughly handled; but not one of these accordances is to be generally observed: while, on the contrary, in all states of the wound, where the operation has been very severe, where the exposed gut has been most handled and squeezed during the operation, I have frequently known no peritoneal inflammation follow; and, where a patient dies of this *secondary* peritoneal inflammation (if I may so call it), the intestine that has been included in the sac, and handled frequently, bears signs of a less degree of inflammation than it exhibited at the time of the operation; or it appears to have been even less inflamed and injured than the gut above.

But now I have almost always seen this peritoneal inflammation, both in its degree and in its progress, manifestly bearing a direct proportion to the severity of the symptoms previous to the operation, and relieved by it,—to the length of time those previous symptoms, and to the length of time the mere strangulation, were allowed to continue; and, lastly, to the debility that has been induced by means employed as auxiliaries to the taxis.

Must it not, then, be hence concluded, that this peritoneal inflammation, although coming on from twelve to twenty-four hours after the operation, is not commonly its effect, but that of the previous strangulation itself, and of means ineffectually, and therefore injuriously, employed in the first instance for its relief? or, indeed, can any other conviction be entertained?—

the fact I formerly stated being borne in mind, viz. that wherever I have seen the operation performed before any symptoms had come on, excepting those essential to every case of strangulated hernia, the wound has healed as well as its magnitude permitted us to hope; and the patient has recovered as rapidly as his constitution allowed us to expect.

From among many in his possession, the author here introduces but two cases; the comparison of which will illustrate, perhaps as well as two cases may, the opinions he has submitted.

Case I.—Mrs. B. a delicate lady, æt. sixty, of extremely retired habits, and almost morbid sensibility, had for many years an irreducible femoral hernia on the left side. It had been more than once apparently strangulated, but had, happily, been always reduced by perseverance in the taxis; the lady declaring, in every instance, that she was willing to die, but not to submit to an operation. In October last, her bowels having been obstructed for two days, the tumor, which was about the size of a goose's egg, became painful, and the patient awoke with violent pain in the region of the stomach. In four hours the surgeon saw her. He found her countenance pallid, expressive of extreme despondency and distress; her mind, indeed, seemed impressed by a confidence of the immediate approach of death; her pulse was small, hard, and frequent; the tumor exceedingly incompressible and tender; any pressure upon it, or upon the abdomen, within a distance of six inches from the groin, producing excruciating pain. She was ordered to take ol. ricini ℥j. secundâ quâque horâ; she lost sixteen ounces of blood from the arm; cold was applied to the tumor; such attempts as were considered justifiable were made to return the intestine; and they were sufficient to show that the operation alone afforded a prospect of success. It was, therefore, performed seven hours after the surgeon first saw her, and about forty-eight hours after the constipation commenced.

Upon opening the sac, a fold of the small intestines was discovered, surrounded by a mass of adhering omentum. The gut presented a healthy appearance. Its return was attempted now that the integuments were from off it; but the division of the stricture was found absolutely necessary. The intestine was at length returned, the omentum remaining in the sac. The edges of the wound were approximated, and slightly dressed; saline purgatives, with powder of cochineal, were prescribed. The violence of the pain in the region of the stomach ceased almost immediately; the tenderness around the tumor speedily abated; the patient became calm and composed. She had stools in the evening, coloured by the cochineal. The external wound healed by the first intention; and, though it afterwards required to be opened for the evacuation of pus from the sac, the patient went into the country, and the wound completely healed in five weeks.

Case II.—Sarah P. æt. sixty, (in appearance, however, rather older,) had had a femoral hernia for six years. During the last three months it had been, on several occasions, irreducible, until after from ten to twelve hours, by the aid of fomentation, it had always ultimately gone up without a surgeon's assistance. In December last, the hernial tumor became much larger than usual, and was irreducible. The patient, however, had a motion on the following day; after which she became sick, and her abdomen became slightly tender. When the surgeon saw her, she had been forty-eight hours without a stool; there was no tension of the belly, and but slight tenderness; she did not vomit. The tumor appeared to contain omentum, by the side of which a small portion of the intestines seemed to have been squeezed down. The taxis was tried, and failed; the swelling remaining tense. The patient was kept in the warm bath till she was faint; she was afterwards bled to sixteen ounces; a purgative enema was administered, and cold was applied to the tumor. The injection returned without feculent matter, at noon (of the third day), and she vomited twice a dark-coloured fluid. On the next day, (the fourth, inclusive, from the time of the hernia becoming irreducible, and the third from her having a stool,) she appeared in all respects as well as on the preceding day.

On laying open the hernial sac, its contents appeared such as had been supposed. The omentum, being adherent, was not returned. A saline purgative was ordered to be taken every two hours; and in five minutes a copious evacuation was produced. The tenderness of the abdomen abated; the patient passed a comfortable night; and in the morning was, to all appearance, as well as could have been hoped.—The purgative to be repeated every sixth hour.

On the second day from the period of the operation, she had a restless night; her abdomen was tender; her bowels had been confined for twelve hours; pulse ninety; tongue foul, and dry. These symptoms increased; but were afterwards remitted during the night, upon her bowels being freely opened.

On the fourth day from the time of the operation, the ligatures, with which the wound had been closed, had broken away; the edges of the wound had separated. The discharge was sanious; the omentum was gangrenous and protruding; the pulse hard and quick, but the bowels still open. She was prescribed cinchona.

From this time she got worse: her bowels becoming obstinately costive; the pulse weak; the tongue furred in the centre; the stomach rejecting its contents; and the omentum sloughing. But, on the ninth day after the operation, the wound looked clean; and the stimulants, with the opium that she had taken, appeared to have had a beneficial effect. On the tenth day, however, cold sweats and delirium came on; the wound was again sloughy; and the woman died of gangrene next morning.

Upon dissection, that portion of intestine which had been in the hernial sac was dark coloured, but not apparently gangrenous; and the omentum was found mortified to some extent within the abdomen.

The treatment in this latter case was not severe. As far as the histories of the two cases prior to the operations are compared, in the delay alone it differs from the former; yet how opposite the events.

The bad consequences of continued strangulation of an intestine, are not the only inducements to operate, as soon as reduction by the taxis appears hopeless. If the operation have its dangers and objections, the taxis is not always free from risk. Wherever strangulation has taken place, and the surgeon cannot return the intestine, but finds it incompressible, and in an inflamed neighbourhood, the case is complete; and, for all that can be discovered in the taxis, the gut may be so gangrenous as to burst; and I strongly suspect I have known it burst, even under gentle and justifiable efforts at reduction;—or again, as I have seen in two instances, it may have been cut or ulcerated through by the sharp edge of a very tight stricture;—or again, the intestine may be strangulated in a portion of omentum, and in such a state returned by the taxis; or, lastly, as I have also known, the gut may be only incarcerated, and so capable of being emptied by the taxis into the intestine above or below, without being capable of liberation sufficient to prevent constipation, and even gangrene, of the bowels above.

Upon the above considerations, therefore, the author has been led to distinguish those cases in which it is not likely that delay can possibly be attended with advantage. He has noticed some wherein the intestine is to be felt through the integuments to be hard, distended with feculent matter, and as if adherent; others wherein the gut appears turned up over Poupart's ligament, suddenly bent on itself or on the stricture, and strictly confined by the circumjacent parts;—others again where the protruded portion of intestine is so small, as to be evidently unmanageable; or so enveloped in omentum, or so restrained, as to preclude all hope of successfully applying the taxis;—others again wherein, the hernial tumor becoming incompressible, its contents are so little distinguishable by the touch, as to warrant the opinion that they will be confined yet more, and are already confined too strictly to admit of successful treatment without an operation;—others again wherein strangulation has already continued long enough to have induced a degree of peritoneal inflammation, prospectively dangerous;—and, lastly, others wherein those symptoms, easily to be overlooked, are appearing, which indicate that fearful torpor of the restorative powers, that renders immediate and complete relief the patient's only chance of life.

In such cases as these, therefore, which I myself have observed, and in all others that are comparatively desperate, or excessively doubtful as to their event, after a competent judge has been consulted, and after he has decided that it is next to impossible, or highly improbable, that the taxis, however aided by treatment, will prove successful,—I submit it to such an one, and I beg to submit it to the reader, whether the chance of having performed an operation, comparatively so trivial, without absolute necessity, be not preferable to that of having employed means, inevitably so severe, without any relief?—whether the patient's chance of recovery, and even of a permanent cure, by an immediate operation, while he is comparatively in health, be not more than what he will retain if the operation be procrastinated,—if it be preceded by a process as severely debilitating as the time will permit?

*South Audley-street; Oct. 12th,* 1824.

